# Novel Substrates as Sources of Ancient DNA: Prospects and Hurdles

**DOI:** 10.3390/genes8070180

**Published:** 2017-07-13

**Authors:** Eleanor Joan Green, Camilla F. Speller

**Affiliations:** BioArCh, Department of Archaeology, University of York, Wentworth Way, York YO10 5DD, UK; eg715@york.ac.uk

**Keywords:** ancient DNA, methodological advances, PCR, NGS

## Abstract

Following the discovery in the late 1980s that hard tissues such as bones and teeth preserve genetic information, the field of ancient DNA analysis has typically concentrated upon these substrates. The onset of high-throughput sequencing, combined with optimized DNA recovery methods, has enabled the analysis of a myriad of ancient species and specimens worldwide, dating back to the Middle Pleistocene. Despite the growing sophistication of analytical techniques, the genetic analysis of substrates other than bone and dentine remain comparatively “novel”. Here, we review analyses of other biological substrates which offer great potential for elucidating phylogenetic relationships, paleoenvironments, and microbial ecosystems including (1) archaeological artifacts and ecofacts; (2) calcified and/or mineralized biological deposits; and (3) biological and cultural archives. We conclude that there is a pressing need for more refined models of DNA preservation and bespoke tools for DNA extraction and analysis to authenticate and maximize the utility of the data obtained. With such tools in place the potential for neglected or underexploited substrates to provide a unique insight into phylogenetics, microbial evolution and evolutionary processes will be realized.

## 1. Introduction

The first successful recovery of ancient DNA (aDNA) in 1984 was from a piece of dried muscle, still connected to the salt-preserved skin of a quagga (*Equus quagga*), which had died ~140 years previously [1]. The demonstration that mitochondrial DNA (mtDNA) could be isolated and cloned from museum specimens influenced the choice of ancient samples for the next decade. Early aDNA research focused on soft tissue [2,3,4,5,6,7,8], however, the first successful extraction of aDNA from archaeological bones by three independent laboratories in 1989–1990 revolutionized the field [9,10,11]. The ability to amplify DNA from (sub)fossils gave-way to a new branch of aDNA studies; paleo-geneticists no longer focused their attention solely on relatively rare soft tissue specimens, but instead began exploiting the component of a vertebrate’s body most likely to survive through time: bone.

**Figure 1:**
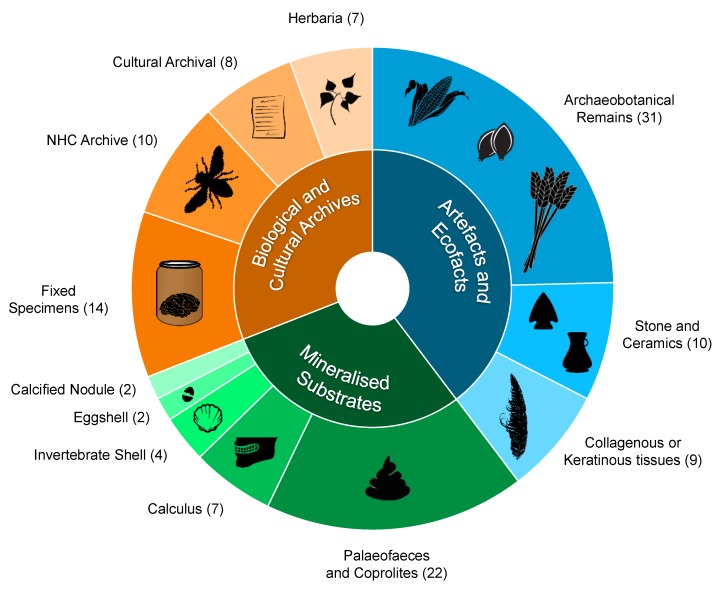
The relative proportion of studies discussed in this review (126 papers from 1988 to May 2017) targeting various alternative substrates for the recovery of ancient DNA. NHC: natural history collections.

For the last 30 years, bone and teeth have been the most frequently studied substrates in paleogenetic research. Most paleontological hard tissues, however, contain very low proportions of endogenous DNA [12], and thus the search for methods to maximize the utility of endogenous DNA data from bony specimens has been extensive, including rapid column-based extractions [13,14,15], and the use of alternative skeletal elements (e.g., the petrous bone, tooth cementum [16,17,18,19]). However, it has been the development of massive parallel DNA sequencing (so called “next generation sequencing” or NGS), often coupled with enriched capture-based methods [12,20] that have resolved two fundamental limiting factors of aDNA research: cost and time. With improved accessibility to highly decayed and fragmentary genetic information, facilitated by the NGS revolution, aDNA is increasingly being extracted from what are now considered novel or alternative substrates (Figure 1).

## 2. Novel Substrates

Although all biological materials have the potential to preserve DNA, relatively few will resist decay over time, unless protected within an inorganic matrix or deliberately preserved through human intervention. Here, we review the potential for DNA preservation within three broad classes of materials: (1) archaeological artifacts and ecofacts; (2) calcified or mineralized substrates; and (3) biological and cultural archives. 

### 2.1. Archaeological Artifacts and Ecofacts

Archaeological excavations worldwide recover millions of artifacts drawn from all time periods in all stages of decay—yet the only materials routinely analyzed genetically are hard tissues. Bones and teeth are mostly targeted due to scientific focus on human (and, to a lesser extent, animal) evolution, while the extraction of DNA from other hard tissues like preserved antler, or antler artifacts has facilitated research into cervid population genetics and object manufacture [21,22]. Researchers are now extracting DNA from an array of artifacts (i.e., objects manufactured by human beings) and ecofacts (i.e., organic materials recovered from archaeological sites that carry anthropological significance) including archaeobotanical remains (seeds, wood, worked plant remains), keratinous and collagenous tissues (feathers, hair, nail and leather), and lithics and ceramics. Although DNA preservation is variable, these items provide unique access to evolutionary pathways, taxonomy and phylogeny that may be simply unobtainable from the analysis of hard tissue.

#### 2.1.1. Archaeobotanical Remains

The genetic analysis of archaeobotanical specimens is relatively well established, and thus no longer “novel”; however, compared to vertebrates, archaeobotanical studies remain vastly underrepresented. A range of early polymerase chain reaction (PCR) based approaches (e.g., [23,24,25,26,27,28,29]) and more recent whole genome studies (e.g., [30,31,32,33,34,35]) have demonstrated the potential to access genetic information held within preserved plant remains. Seeds tend to preserve in archaeological sites only when charred, desiccated, frozen, or deposited in anoxic conditions. Charred seeds, making up the vast majority of recovered archaeobotanical materials, have variable degrees of DNA preservation, depending, in part, on the extent of charring, as well as their age and depositional environment [36,37,38]. For example, in Bunning et al.’s [30] analysis of 3000 year-old charred, mixed cereals from Assiros Toumba (Greece), less than 0.1% of the over 21 M recovered sequences could be assigned to the kingdom Viridiplantae. Likewise, Nistelberger et al. [34] obtained 0–0.12% endogenous DNA from 4450 to 550 year-old charred barley, grape seeds, maize cobs, and rice remains from a range of contexts worldwide, even using a targeted enrichment approach. Variation in DNA preservation is also observed within desiccated remains. For example, in their analysis of five 6000 year-old barley grains from a single site in Israel, Mascher et al. [33] reported endogenous DNA ranging from 0.4 to 96.4%. 

Successful DNA recovery has also been achieved using other archaeological plant tissues, including maize cobs [32,39,40], fruit stones [41], pollen [42,43,44], grains and seeds [45,46,47,48], rind [37], and chaff [49,50]. Plant-based artifacts can also be analyzed for their human DNA content—for example, LeBlanc et al. [51] extracted DNA from quids (chewed plant material) and fiber aprons (sanitary wear) to identify human mtDNA haplotypes from Southwest America. Despite PCR methods suggesting there were few prospects for retrieving aDNA from wood [52,53] due to PCR-inhibiting metabolites [54,55], recently the application of NGS to ~9000 year-old archaeological wood has recovered DNA fragments of 87–596 base pairs (bp) [56]. Using more advanced DNA recovery and bioinformatic techniques such as whole genome sequencing, single nucleotide polymorphism (SNP) capture, exome analyses, RNA analysis, and methylation patterns, these archaeobotanical projects are demonstrating the vast potential of ancient plant remains to address significant debates around the origins, movement and adaptation of domestic crops [32,37], reconstruction of paleo-environments [57] and models of DNA decay within plant remains [34,36].

#### 2.1.2. Keratinous and Collagenous Tissues

Leather, hair, baleen, claws and feathers are all composed of layers of collagen or keratin, which frequently decompose when deposited underground. In some extraordinary contexts, (e.g., burial in permafrost), such items may be preserved in the archaeological record and retain host DNA. Although reports on DNA extraction from archaeological leathers are few, mtDNA within this substrate seems to be particularly resilient, with previous studies recovering 70–177 bp fragments from medieval [58] and Neolithic [59] leather; although the tanning process may be particularly damaging for nuclear DNA [58,60]. 

The keratin structure of hair, claws and baleen is thought to protect endogenous DNA from contamination [61,62], and several studies have successfully retrieved endogenous DNA from human and animal hair [62,63,64] and whale baleen [65,66] preserved in Danish and Greenlandic archaeological contexts. Preservation of claws in archaeological contexts is rare; however, research on natural history collections (NHC) indicates that DNA can be sufficiently well preserved for analysis of this substrate [67,68], with potentially the same success rate as ancient bone [69]. The base of feathers (the calamus) has been frequently exploited as a source of high-quality DNA in bird phylogenetics and conservation biology research, and the potential for feathers in NHC to preserve DNA for several hundred years has been known for more than two decades (e.g., [70,71,72]). In archaeological and subfossil environments, the upper shaft and the feather vane are the only part of the feathers that typically survive and were thought to be unsuitable for aDNA analysis. Using Moa feathers preserved in rock shelter sites in New Zealand, Rawlence et al. (2009) demonstrated that both the rachis and barbs, as well as the calamus, can preserve DNA for potentially up to 3000 years. Subsequently, Speller et al. [73] were able to recover mtDNA fragments from as few as two feather barbs, suggesting that feathers may represent a robust source of aDNA when preserved in favorable contexts. 

The potential for mtDNA to survive in these keratinous substrates has been well documented, however, the potential for nuclear DNA survival is less well explored [66,74]. Future metagenomic analyses are required to assess both the relative proportion and survival of mitochondrial versus nuclear DNA, and to test susceptibility of different keratinous substrates to exogenous contamination from the burial environment.

#### 2.1.3. Lithics and Ceramics

Lithics represent some of the most abundant and durable artifacts within the archaeological record, particularly in prehistoric contexts. As such, studies endeavoring to understand the day-to-day use of various stone artifacts have a long history, employing methods such as experimental archaeology [75]; useware analysis [76]; microwear polish examination [77]; and microscopic and chemical residue analysis [78,79]. Unsurprisingly, researchers attempted to extract DNA from lithics as early as the 1990s. Loy [80] reported the identification of bovine satellite DNA from a 2200 year-old stone tool from British Columbia using nested PCR, while Hardy et al. [81] attempted even older samples, reporting the amplification of short fragments of *Sus scrofa* cytochrome b (cytb) mtDNA, from a single stone tool dating to 35,000–65,000 years ago. In 2001, Shanks et al. [82] reported observation of fluorescently labeled blood protein and DNA within the microcracks of stone tools, and later [83] the purported recovery of 116 bp fragments of cytb mtDNA from these microcracks, the majority corresponding to the canid family. The findings of these early studies have been called into question, first by the lack of authentication measures and contamination controls required to validate aDNA results [84], as well as by observations of domestic animal DNA within laboratory reagents [85]. Coupled with extensive controversy regarding the ability to extract authentic proteins from ancient lithic materials (e.g., [86,87,88,89,90,91]), the potential for stone tools to entrap and preserve DNA had, for many years, seemingly been laid to rest. The application of NGS approaches however, may resurrect this old idea in a new form, again through the analysis of another novel substrate: historic building materials. Two recent studies have investigated the potential for metagenomic analysis of ancient brick and stone work to elucidate building histories and investigate microbial factors influencing biodeterioration of built heritage [92,93], opening up potentially more fruitful research directions for ancient lithics. 

Like lithic materials, ancient ceramics make up a large percentage of archaeological finds; however, attempts to mine this substrate for preserved DNA has lagged significantly compared to the analysis of other surface or adsorbed organic molecules, including lipids, amino acids, alkaloids, waxes, etc. [94,95,96,97]. Attempts to extract DNA from ceramic objects have involved scraping or drilling the interior of the vessel, as well as non-destructive swabbing of the interior face [98,99]. Recently, aDNA identifications have been reported from pottery vessels recovered from environments assumed to be hostile to DNA. Foley et al. [100] reported the amplification of 69–188 bp fragments of plant chloroplast DNA from 5^th^ to 3^rd^ Century B.C. empty amphora recovered from Mediterranean Sea floor, identifying a range of plants known to have been exploited by the Greeks. Based on their results, Foley et al. claimed that sufficient DNA could be recovered merely by swabbing the inner surfaces of ceramic fragments that had been lying beneath the ocean floor for over two millennia. Robinson et al. [101] also reported the recovery of aDNA from terracotta libation figurines from pre-colonial Ghana using a swabbing approach. Here, DNA from three plant types—plantain, pine and grasses—were identified using generic plant primers, recovering fragments up to 257 bp, significantly beyond the average length of DNA fragments they predicted by thermal age modeling. The latter study applied extensive contamination controls and numerous measures to validate their sequences, but, in so doing, highlighted a major challenge when utilizing atypical substrates: due to the infancy of the research, the proper authentication of results is imperative; paradoxically, there is currently little understanding of if and how DNA is expected to preserve within such substrates—an issue we discuss in greater detail below (Section 3.1).

### 2.2. Calcified and Mineralized Substrates

The predominant use of skeletal hard tissues in aDNA analysis is related to their ubiquity in the archaeological and paleontological record, but also because the inorganic fraction of hydroxyapatite in bone and teeth is thought to stabilize and preserve DNA through adsorption [102,103]. More recently, the potential for other mineralized substrates to preserve ancient biomolecules in deep time is also being realized [104], including substrates such as archaeological dental calculus, coprolites, calcified soft tissues, invertebrate shells and ancient eggshells. Unlike bones and teeth, these substrates may be exploited not only as a source of host genetic information, but also to provide insight into ancient microbiomes and paleoenvironments. 

#### 2.2.1. Calculus

Dental calculus (tartar) is a bacterial biofilm composed from dental plaque, saliva and gingival crevicular fluid, mineralized within a matrix of multiple calcium phosphates, forming a cement-like substrate on the surface of teeth [105,106,107]. Although found virtually ubiquitously on archaeological human skeletons without modern dental hygiene interventions, the potential for this substrate to preserve abundant and varied ancient biomolecules has only recently been discovered. The preservation of ancient oral bacterial DNA was first recognized through gold-labeled antibody transmission electron microscopy [108] and subsequently through targeted PCR and 16S ribosomal RNA (rRNA) amplicon metagenomics [108,109,110]. Shotgun metagenomic approaches have demonstrated that although calculus is dominated by bacterial (and to a lesser extent fungal, archeal and viral) DNA derived from the oral microbiome, minute quantities of host DNA as well as inhaled or ingested eukaryotic DNA also survive [111,112,113]. Research to date has demonstrated that calculus preserves an exceptional abundance of entrapped DNA and proteins [109,112,113,114], and even ancient metabolites [115]. These combined studies have provided insight into the oral ecology of ancient humans and hominids, and even allowed for microbial genome reconstruction dating back to nearly 50k years before present (BP) [112,113]. In some studies, calculus has been noted to be relatively resistant to exogenous bacterial colonization from the burial environment [113]. In combination, this recent work highlights the promise of calculus as a “novel” substrate for reconstructing ancient microbial genomes, tracking evolutionary changes in oral ecology, detecting dietary and environmental information, and analyzing host population demographics through mitochondrial genome capture [105,116,117].

#### 2.2.2. Paleofeces and Coprolites

Unlike calculus, paleofeces and coprolites have been long been recognized for their value as a source of ancient biomolecules and paleodietary information. Paleofeces can be preserved via various mechanisms such as rapid desiccation or waterlogging, while coprolites form through the precipitation of calcium and phosphate from ingested bone (especially in carnivores and scavengers) or through the acquisition of preservational constituents via groundwater [118]. Due to the specific environmental conditions required for their preservation, paleofeces and coprolites are also relatively rare archaeological finds compared with the ubiquity of dental calculus. At the turn of the millennium, Poinar et al. [119,120,121] and Hofreiter et al. [122] first demonstrated the molecular potential of coprolites by amplifying and cloning DNA from extinct ground sloth and human coprolites to confirm the depositing species, and to investigate the diversity of ingested plant and animal material. These studies laid the foundation for other human and animal paleofeces and coprolite analyses using targeted PCR to identify the presence and genetic diversity of humans in the Americas [123,124,125], elucidate the taxonomy and diet of extinct [126,127,128,129] and extant birds [130,131] as well as to taxonomically identify parasite eggs within coprolites [132,133,134]. 

More recently, high-throughput approaches have been applied to recover more representative observations of dietary components within paleofeces and coprolites. Last year, Wood et al. [135] applied metabarcoding of eukaryotic 18S rDNA and plant chloroplast *trnL* to investigate the diet of Polynesian domestic dogs, identifying the main dietary components as marine bony fishes (*Euteleosteomorpha*) and plants (*Cucurbitaceae*). Analysis of retroviral DNA has also been attempted to access paleodietary information. In their metagenomic analysis of ancient human coprolites from the Caribbean, Rivera-Perez et al. [136] reported a range of eukaryotic retroviruses through a BLASTX comparison of the non-redundant NCBI database. Although the identified retroviruses corresponded with expected dietary patterns within the region, the dominance of well-characterized taxa (e.g., fowlpox virus) and model organisms (e.g., *Xenopus*) within the identified sequences, coupled with the detection of viruses infecting European plants (e.g., *Morus notabilis*) in pre-Columbian contexts raises questions about the extent to which the quantity and quality of reference sequences within public databases may bias the identifications (see “Section 3.2”). 

Paleofeces and coprolites, however, provide genetic information beyond dietary inclusions. Tito et al. [137] were the first to recognize that coprolites and desiccated feces can facilitate the study of ancient gut microbiomes by reconstructing the microbial ecology of two 1300 year-old coprolites from Mexico through shotgun metagenomics. In a follow up study using a 16S rRNA metabarcoding approach on an expanded sample set, they revealed that not all fecal deposits may preserve the signature of endogenous ecology at the time of deposition [138]. While rapidly desiccated feces from cave environments were found to preserve the integrity of the gut ecology, others were found to have undergone self-digestion and decomposition as well as bacterial infiltration from the burial environment. The portion of coprolite sampled for analysis can also bias the identified microbial communities. Cano et al. [139] noted in their study of human coprolites from Central America that larger proportions of soil microbes were observed within the cortices compared to the inner core, likely reflecting environmental contamination on the coprolites surface; subsequently Wood and Wilmshurst [140] have recommended subsampling protocols to target the coprolite core. Paleofeces and coprolites can also reveal the presence of ancient viruses. Multiple-displacement amplification of viral genomes within a 14^th^ Century human coprolite revealed an ancient virome comprised predominantly of double-stranded viral DNA (85.21%), with the majority of identifiable sequences corresponding to bacteriophages, especially *Siphoviridae* [141]. Although the virome was similar to that found in modern fecal matter and soil, further characterization of virome taxonomy and function was limited by the relatively high proportion of sequences of unknown origin.

#### 2.2.3. Calcified Nodules

The human body may produce a variety of calcified tissues or “biological stones” with highly diverse structures and chemical compositions [142,143]. Although found principally within the digestive or urinary tracts, calcified tissues and stony neoplastic tumors or exudates may form within the lungs, vascular system, tear ducts, tendons, or skin, and are occasionally recovered during archaeological excavations. Two recent studies have exemplified the wealth of genetic information that may be preserved within these serendipitous finds. Kay et al. [144] applied metagenomic analysis to a calcified nodule recovered from the pelvic girdle of a 14^th^ Century male skeleton from Geridu (Sardinia). Although tuberculosis was initially suspected as the cause of the pathology, the study instead identified sequences matching *Brucella melitensis* (0.48%), ultimately obtaining sufficient sequences to allow the reconstruction of a complete genome with 6.5× coverage. The proportion of human DNA within the nodule was relatively high for archaeological material (23%), demonstrating the potential of these substrates to provide information both on host genetics and bacterial pathogens. In a subsequent study, scanning electron microscopy and metagenomic analysis were used to investigate calcified abscesses recovered from a woman’s skeletal remains at a Byzantine cemetery in Troy [145]. Microscopic analysis revealed images of “ghost cells” preserved in a mineralized layer resulting from dystrophic calcification. Like the calcified nodule from Sardinia, the tumor contained an amalgam of human and microbial DNA. The human sex chromosome component of the DNA revealed a dominance of X chromosome sequences, with minute quantities of conserved Y chromosome sequences, interpreted to result from the presence of a male fetus, and suggesting that the growths developed in placental tissue [145]. The bacterial DNA recovered from shotgun sequencing identified *Staphylococcus saprophyticus* and *Gardnerella vaginalis* in high abundance, allowing for genome reconstruction and strain level analysis. Although calcified nodules may represent a rather unusual archaeological find, these case studies display the potential of these substrates to provide insight both into host genetics and ancient health and disease.

#### 2.2.4. Invertebrate Shell

There is a long history of research around organic molecules preserved within the calcareous exoskeleton of marine invertebrates. Previous research has focused almost exclusively on the preservation of proteins, particularly those responsible for biomineralization [146,147], and the use of these proteins for amino acid geochronology [148,149,150,151] and more recently, taxonomic identification [152]. The potential for the calcium carbonate of marine invertebrate shell to preserve genetic material has only recently been explored. Pawłowska et al. [153,154] demonstrated that well-preserved DNA could be recovered from micropaleontological taxa, like foraminifera, to reconstruct past micro-eukaryotic diversity and its relationship to climate change. Likewise, Villanea et al. [155] successfully recovered fragments of gastropod DNA from *Naesiotus* shells. Very recently, Der Sarkissian et al. [156] applied high-throughput shotgun DNA sequencing to mollusk shells up to 7000 years old from various countries (France, Korea, Japan, Norway, Italy, Denmark and the UK). They recovered average fragment lengths of 43–50 bp, enabling positive taxonomic identification of various mollusk species, marine organism pathogens as well as minute quantities of mtDNA from the marine environment [156]. Considering the wide array of marine species which produce carbonate exoskeletons (e.g., chitons, gastropods, cephalopods, bivalves, scaphopods) and the ubiquity of such shells in archaeological sites dating back to the Middle Pleistocene [157], there is a huge potential for using these substrates to track long term natural and human-induced changes in marine invertebrate paleodemography and biodiversity.

#### 2.2.5. Ancient Eggshell

Like marine invertebrate shell, eggshell represents another calcified deposit where early biomolecular analysis has focused principally on the protein component to reconstruct both geochronology [158,159] and ancient diets [160], with the genetic potential of ancient eggshell recognized only several years later. In modern genetic studies, as well as investigations on museum specimens, DNA extraction concentrates on the membranous layers on the inner face (e.g., [161,162]), prompting researchers to suggest that aDNA is more likely to survive on this inner shell where the membrane desiccates [163]. Using confocal microscopy to visualize DNA hotspots, Oskam et al. [163] demonstrated that DNA was more heavily concentrated around the boundaries of individual mammillary cones (a layer of the matrix close to the inner eggshell), likely resulting from the incorporation of oviduct epithelial cells into the calcite [164]. Oskam et al. [163] went on to sequence DNA extracted from ancient eggshells successfully conducting quantitative PCR (qPCR) on 16 specimens up to 19,000 years old from New Zealand, Madagascar and Australia, amplifying both mtDNA and a conserved region of the nuclear *c-mos* gene. 

As with all ancient substrates, depositional environments and overall biomolecular preservation seem to influence ancient eggshell DNA quality and quantity. For example, in the sub-tropical climate of Madagascar, targeted enrichment coupled with NGS on elephant bird (*Aepyornis* sp.) eggshell resulted in just 3% endogenous DNA [165]. In spite of the relatively low recovery rate, eggshell DNA preservation may prove to be comparable or even superior to bone. For example, previous DNA analysis from elephant bird bone produced only a partial mitogenome [166]—by comparison eggshell produced a complete mitochondrial genome with an additional 2271 bp, approximately twice the average coverage (33.5×) with fewer than 100 ambiguous sites [165]. Furthermore, Grealy et al. [165] report the recovery of nuclear DNA from eggshell, represented by 12,500 bp of exonic sequences. In their qPCR study of eggshells from various sites, Oskam et al. [163] detected proportionally fewer microbial reads in eggshell (1:10.6) than in bone (1:1333) [163], making eggshell a potentially attractive substrate for investigating avian genetics using high-throughput methods.

### 2.3. Biological and Cultural Archives

Natural history museums house millions of specimens from all over the world, and are the cornerstone of biological and biodiversity research. These collections have been exploited for their rich stores of historic and prehistoric genetic information for decades [167,168], with studies targeting DNA preserved in skeletal tissues, hair [169], claws [67], nails [68], skin [69], and invertebrate tissues [170,171] among others. More recently, other repositories such as herbaria and cultural archives (e.g., anthropological museums, documentary archives) have demonstrated their long-term genetic potential through successful recovery of DNA from historic plant collections [172,173,174,175,176] and parchments [177,178,179]. A comprehensive review of ancient DNA research on all biological and cultural archival material would be outside the scope of this paper; here, we focus on more unusual applications of DNA analysis to NHC, focusing specifically on fixed specimens, which hold particular, yet relatively untapped, promise as long-term reservoirs of ancient genetic material. 

#### Fluid-Preserved Specimens

NHC hold vast numbers of fixed specimens collected over the last ~200 years, preserved typically through immersion in formaldehyde, formalin, methanol or ethanol. Visually, fluid-preserved specimens can appear near-perfect; this aesthetic preservation encouraged attempts at DNA extraction from the late 20^th^ Century, mostly focused on ethanol-preserved specimens [180,181,182,183,184]. Extracting DNA from fixed materials, however, is problematic due to the molecules being particularly prone to oxidative and hydrolytic damage, fragmentation, base modifications and cross-linkage during the fixation process [185,186,187]. Compared with ethanol, formaldehyde and formalin treatments appear to be the most damaging to nucleic acids [188], and extensive research on formaldehyde-fixed clinical tissue specimens demonstrated that even relatively short exposure times (e.g., a few hours) induce DNA degradation [189], reduced DNA solubility [186], decreased PCR success rates due to crosslinking of proteins and DNA [190], and resulted in a higher-frequency of sequence alterations [191]. Ten years ago, the routine recovery of DNA from formalin- or formaldehyde-treated museum specimens seemed unlikely (see review in [192]), though more recent studies have expanded the potential of formalin fixed tissues by elucidating molecular preservation patterns [188], optimizing DNA extraction methods [193,194] and maximizing sequence recovery using NGS approaches [195,196]. 

Fixed specimens offer the potential not only to extract preserved host DNA, but also associated pathogens or even microbiomes. This potential was recognized early by Barnes et al. [181], who attempted to recover both host and pathogen DNA from historic diseased human tissues preserved in ethanol. Although modern biopsies indicated that *Helicobacter pylori* should be present in a ratio of 1:103 compared to human mtDNA, only sporadic evidence of the latter could be recovered from the fixed samples. More recently, Hühns et al. [197] reported success in recovering short fragments of *Mycobacterium tuberculosis* and human papillomavirus DNA from 78 to 12 year-old formalin fixed specimens. With further optimization, sufficient host and pathogenic DNA may be recovered to reconstruct host-pathogen interactions and pathogen evolution over the last several hundred years. Non-invasive approaches may also be an exciting new possibility for these fixed samples. For example, Shokralla et al. [198] have demonstrated the possibility of extracting a specimen’s DNA directly from the preservative ethanol. As extraction and analytical methods continue to evolve, we anticipate exponential growth in the genetic analysis of NHC and the field of “museomics” more broadly [199,200,201].

## 3. Moving Forward

High-throughput sequencing and omic technologies have revolutionized molecular research on ancient samples. With decreasing costs and increasing capacity, we have witnessed a surge of studies applying these techniques to a range of atypical archaeological materials and historical collections. Although the analysis of unusual biological substrates has the potential to open exciting new research directions in paleontology, paleoecology and paleogenetics, there are a number of key ethical and methodological considerations that need to be addressed if their analysis is to move beyond “novelty” to make lasting scientific contributions. Many of these new technologies have been applied to rare archaeological or paleontological finds, and there are significant ethical issues associated with the destructive analysis of finite archaeological or historical resources. First and foremost, researchers have an obligation to apply destructive techniques only if there is deemed a high likelihood of analytical success. However, our knowledge is limited on how genetic information may be preserved within these different materials. Furthermore, these atypical substrates may require specialized extraction techniques or analytical approaches to maximize the information that can be obtained from these precious resources. Below, we discuss issues of DNA preservation, authentication and analytical approaches in more detail, and make some recommendations for areas of future research to maximize the paleogenetic data that can be obtained from these varied resources. 

### 3.1. Predicting DNA Survival

To date, much of our understanding of DNA preservation in ancient substrates has been limited to hard tissues, such as tooth and bone [202,203,204,205]. Although it is expected that DNA within all ancient substrates will be highly fragmented [204], enriched in GC content [206], and feature mis-incorporations due to cytosine deamination and single-stranded fragment overhangs [207], we have little understanding of the extent to which degradation processes will limit analytical success in various biological substrates, especially considering the wide range of environments in which they have been preserved. In addition to confounding variables of time and temperature, archaeological artifacts and NHC may have been subjected to processing (e.g., tanning or dyeing) and/or conservation treatments that may also influence DNA degradation [58,188,208,209,210]. 

The majority of models predicting DNA degradation and fragmentation have been based on ancient bone. For example, decision-making models such as thermal-age.eu [211] have been developed to predict DNA preservation based on temperature-dependent rates of depurination in ancient bone [203]. Subsequent modeling of DNA preservation in previously published hard tissues genomic datasets suggested that while cytosine deamination is affected by sample age and the temperature of the depositional environment, DNA fragmentation rates do not appear to be influenced by age [202]. Instead of a constant rate of degradation, multiple stages of decay occur. Beginning with an intense initial stage of microbial attack and cellular activity that results in the destruction of significant quantities of endogenous DNA, a threshold is eventually reached, whereupon the DNA appears to be relatively stable, provided that the depositional environment is conducive to preservation [202]. This dual model of fragmentation has also been observed in anthropogenically preserved NHC specimens [188] and herbarium collections [209], suggesting that even tissues collected within the last century will display significant DNA degradation that may influence analytical success. Together, these findings suggest that DNA decay does not operate on a simple linear scale, and predicting the ultimate decay pattern in individual samples involves the detailed consideration of multiple complex processes resulting from primary depositional environments and/or conservation and subsequent storage. 

The extent to which DNA preservation mirrors that of other biomolecules is also a key concern. Proteins survive longer than DNA in the archaeological record, but the extent to which proteins can be used to predict DNA survival is still under debate [212,213,214,215]. Alternative substrates such as archaeological dental calculus [113,114], and food residues [216] have yielded some of the most abundant ancient proteomes, while Demarchi et al. [104] demonstrated the persistence of eggshell proteins as far back as 3.8 million years. The remarkable survival of these molecules is thought to result from protein binding strongly to a calcite surface, preventing hydrolytic breakdown. The extent to which similar factors may influence DNA survival in eggshells, and other calcified deposits, warrants further systematic testing to maximize the potential of these substrates. Understanding the methods by which DNA may bind to non-organic substrates like pottery or lithics is of particular importance, as we currently have little experimental data upon which to build reliable degradation models. Experimental methods, involving artificial DNA degradation [36,217,218,219,220,221] and different specimen treatments [58,64,88,210,222], are essential for developing more nuanced and informed models of DNA preservation on various substrates. These systematic studies are essential not only for making more informed choices when selecting rare samples for analysis, but for providing the necessary data to authenticate ancient genetic sequences recovered from atypical samples.

### 3.2. Authenticating Results

Authenticating endogenous DNA is a longstanding challenge faced by those studying aDNA, with multiple authentication criteria put in place and elaborated over time [84,223,224,225,226,227,228]. As the field advanced in terms of our understanding of DNA degradation, so too has our ability to recognize contaminating sequence by their deviation from “expected” molecular behavior. While the pre-2008 era of targeted PCR and Sanger sequencing relied principally on the criteria of authenticity proposed by Poinar and Cooper [84], more recent NGS studies have relied primarily on demonstrating “typical” signatures of post-mortem damage (PMD), principally cytosine deamination at 5’-overhangs [207,229] and sequence length distribution plots demonstrating DNA fragmented by hydrolysis. Detection of these expected patterns is now facilitated by the application of software such as “mapDamage” [230,231] and “bamdamage” [232], which quantify C-T transitions at the 5’ end and G-A transitions at the 3’ end of sequences. Over the last few years, researchers have come to rely heavily on damage plotting software as the primary means of authenticating data, even when studying unusual substrates where our knowledge of DNA degradation patterns is lacking. For example, Kay et al. [144] applied a damage pattern assessment in their metagenomic analysis of a 14^th^ Century calcified nodule identifying the expected PMD pattern, but also discarded any sequences >150 bp (regardless of PMD). Although their comprehensive controls and conservative approach to data authentication is to be commended, the lack of knowledge of “appropriate molecular behavior” in novel substrates means that we may be potentially discarding authentic genetic data by assuming degradation patterns identical to those of bone or teeth. 

A second challenge posed by unconventional substrates is that, unlike skeletal tissue, the taxonomic identity of the source(s) of the DNA (i.e., the species) is often unknown, for example in the metagenomic analysis of coprolites and calculus, or the analysis of artifacts, and thus more difficult to validate. Many of the aforementioned studies have used the BLAST application as a means of taxonomic identification for either metagenomic [34,136] or targeted amplicon sequences [101,135]. Although BLAST searches against the NCBI database is a widely used method for detecting sequence homology [233], taxonomic misidentification is a distinct possibility when working with short, damaged ancient DNA fragments. When studying ancient samples this problem is heightened since for many extinct species there are no well-matched modern genomes that could be used for comparisons [234]. NCBI entries also contain many low quality sequences that have not undergone full annotation and robust quality checks [235], and genomic sequences may include adaptor sequences [236], as well as contaminant human [237], domestic animal [238] and bacterial reads [239], which may result in query sequences being falsely aligned to non-authentic species. This is a particular challenge for microbial analysis, where closely related environmental contaminants and false positive matches against pathogens can easily confound taxonomic identifications [240]. Although using validated, curated databases of reference sequences (e.g., NCBI RefSeq) may be a more secure approach to taxonomic identification, species identifications resulting from low-coverage data should always be approached with caution. 

We agree with Carl Sagan that “extraordinary claims require extraordinary evidence”; until the analysis of atypical substrates becomes more routine, the results require an additional burden of proof for authentication. Many of the early authentication criteria, such as physically isolated laboratories, quantitation, detection of hydrolytic and oxidative damage and independent replication remain applicable to novel substrates [228], although these are never a substitute for critical thought [227]. As with all types of scientific inquiry, multiple lines of evidence are more powerful than one, and the incorporation of multiple biomolecular approaches serves not only to validate the results, but maximize the data that can be obtained from these, often extraordinary, materials.

### 3.3. Maximizing Information

Researchers have an obligation to maximize the data obtained during destructive analysis, but particularly when working with rare or novel specimens. Factors such as sample size, extraction method, sequencing approach and bioinformatic analysis are all important considerations for increasing the amount of retrieved data, while reducing the destructive impact of sampling.

#### 3.3.1. Sampling and DNA Extraction Methods

As most of the ancient genetic work of the last 30 years has been conducted on bone and teeth, sampling protocols and extraction methods have been tailored accordingly to maximize the retrieval of endogenous DNA [14,15,16,241,242]. With our limited understanding of where and how DNA preserves in non-bone material, there is pressing need for the development and systematic optimization of DNA extraction protocols from a variety of other tissues. The importance of bespoke protocols has recently been demonstrated for herbaria; for example, Weiß et al. [174] estimated that herbaria specimens have a per-nucleotide fragmentation rate of 1.66 × 10^−4^ meaning that DNA from even relatively recent herbaria specimens is expected to be highly fragmented. Gutaker et al. [175] subsequently modified their herbaria extraction procedure by using a *N*-phenacylthiazolium bromide buffer and altering the binding conditions of DNA to obtain a greater proportion of ultrashort (<50 bp) DNA fragments. Although over the last few years, some studies have begun to investigate the efficacy of different sampling and extraction protocols for degraded plants [243,244,245], archaeological dental calculus [113], coprolites [125,140], parchment [179] and formalin-fixed tissues [193,246], these have typically been conducted only when sufficient materials are available for systematic analysis. For more rare materials, such as archaeological artifacts, systematic testing may rely more on modern experimental or artificially degraded materials. 

The difficulties in authenticating aDNA from atypical substrates, and the desire to maximize information from these unique genetic resources provides a compelling case for “unified protocols” or multi-proxy approaches [140,247] that are capable of extracting multiple biomolecules or organic residues from the same piece of starting material. The aforementioned studies have demonstrated a range of analytical approaches that can both complement and validate ancient genetic information, including microscopy [113,135], lipid analysis [248], proteomics [113,249], metabolomics [93,115], radiocarbon dating [122,128], collagen peptide mass fingerprinting [22], etc. In the majority of these studies, these complementary approaches have required the destruction of additional material; we advocate that future studies attempt to co-extract and analyze multiple biomolecules. 

Non-destructive and non-invasive sampling methodologies are increasingly being demanded by museums and archives in order to preserve the integrity of unique accessions and maintain sufficient material for future analyses [250,251,252]. A variety of studies have developed novel biomolecular sampling techniques for bones and teeth [253,254], insects [170,255,256], shells [152], books and parchment [249,257], and fluid-preserved specimens [198] which do not require invasive sampling. As both our extraction techniques and bioinformatic approaches improve, potentially greater amounts of information may be achieved from minute samples.

#### 3.3.2. Sequencing Technologies and Analytical Approaches

**Figure 2:**
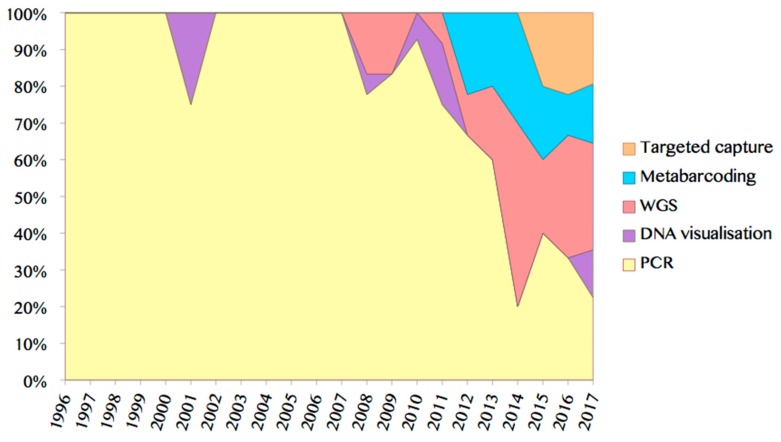
Area chart displaying the proportion of studies utilizing various methods in the analysis of neglected substrates, demonstrating the rise of whole genome and metagenomic approaches following the emergence of NGS technologies (includes studies reviewed in this paper, from 1996 to May 2017). WGS: whole genome sequencing; PCR: polymerase chain reaction.

While early studies obtained ancient genetic sequences through PCR coupled with Sanger sequencing, since 2010 there has been a greater push towards metabarcoding and whole genome approaches coupled with high-throughput sequencing (Figure 2). Here again, bespoke analytical methods may vastly improve our access to ancient genetic information particularly when studying unusual substrates that are suspected to preserve only highly fragmented DNA. For example, Gansauge et al. [258] and Stiller et al. [259] both recently noted significant improvements in library yields from formalin-fixed tissues using a single-stranded vs. a double stranded library preparation method. Similarly, Gómez-Zeledón et al. [56] achieved success in recovering informative SNPs from highly fragmentary and inhibited archaeological wood samples using optimized TaqMan qPCR assays. 

The endogenous DNA content of ancient materials is variable to say the least, and may represent 0% of the total DNA recovered in whole genome approaches. As a response, targeted enrichment or “capture” based methods, where DNA of interest is immobilized on custom-designed baits in solution or on a physical array, is emerging as a more frequent methodological approach for atypical substrates, and has, for example, been applied to archaeological dental calculus [111], ancient eggshells [165], charred archaeobotanical remains [34] and fluid-preserved specimens [195] in order to recover mtDNA genomes, enrich full genome coverage, or capture ultraconserved elements, respectively (Figure 2).

Novel substrates, however, are increasingly being recognized for the genetic information they provide, not only about the host, but also associated microbes and microbiomes. While the sequencing of endogenous DNA facilitates the study of host genetics including ancestry, provenancing, phylogenetic and evolutionary questions (e.g., [260,261]), it is only one aspect of the biological history of an organism. As host microbes outnumber endogenous host cells at a ratio of at least 2:1 [262], and considering that humans (and animals) are critically dependent on these >1000 bacterial species, focusing entirely on the host genome means that an entire ancient ecosystem is disregarded [263,264]. Shotgun sequencing approaches to coprolites, calculus, calcified nodules, and shells have already demonstrated the ancient microbial information that may be obtained from non-skeletal tissues, in some cases allowing for reconstructions of complete ancient microbial genomes [112,113], demonstrating the vast potential of these substrates to reconstruct the evolution of host-microbe interactions. For example, shotgun sequencing of ancient eggshell may reveal evolutionary changes in eggshell microbiomes, albumen immune defenses and avian pathogens that influence egg mortality [265], while metagenomic sequencing of fluid-preserved specimens may allow for the reconstruction of myriad vertebrate microbiomes in various states of health and disease. Metagenomic approaches may also provide information on the conservation status of museum collections and artifacts [92], for example, by revealing microbes implicated in their deterioration and decay [266]. 

The move towards whole genome approaches and NGS technologies yields significantly more data than have traditional sequencing methods, with much of this data made up of increasingly short DNA fragments. As bioinformatic methods improve, we will recover ever more genomic and metagenomic information from unconventional substrates, including increasingly high-resolution information on genetic traits underlying local adaptations [29], and phenotypes [267]. A promising advance is the recovery of epigenomic information from tissues other than bones and teeth. The potential of ancient epigenetics has already been demonstrated through the observation of methylation patterns in ancient bison bone [268], human bone [269], and Neanderthal and Denisovan skeletal material [270]. Recently, epigenetics have offered insight into other tissues, such as plant remains and hair [271]. For example, Smith et al. [272] detected genome-wide hypermethylation in ancient barley grains following viral infection, revealing the epigenetic impacts of exposure to biotic stress. The further development of epigenomic recovery and analysis could refine insight into age-at-death for biological organisms [271], provide evidence of how methylation status influenced evolutionary adaptation [270] and elucidate regulatory changes underlying species divergences [273].

## 4. Conclusions

The past three decades of aDNA analysis have witnessed the recovery of genetic material from a wide range of ancient tissues and artifacts, including inorganic matrices once thought to be barren of surviving DNA (e.g., eggshells, ceramics). Further optimization of DNA extraction and analytical techniques, coupled with high-throughput and single-molecule sequencing will catalyze research in this area, allowing greater access to the often low-template, highly damaged DNA retained within these substrates. Nevertheless, these new technology-driven opportunities also present some challenges that need to be addressed, including:

1. Greater understanding of DNA preservation and degradation within various materials and their implications for selecting the most appropriate material for destructive analysis;
2. Developing appropriate multi-proxy approaches for authenticating ancient genetic data;
3. Raising awareness of the common pitfalls of aDNA analysis on atypical substrates that may be unfamiliar to many “modern” genetic laboratories entering the field.

Evidence for progress in these areas is already apparent–over 45% of the studies reviewed above had either method development or modeling DNA degradation as their primary research goal. A richer understanding of the potentials and pitfalls associated with the genetic analysis of these unconventional substrates will ultimately lead to more robust scientific results and more ethically informed decisions on sampling these irreplaceable historical resources.
